# Drug repositioning in metastatic prostate cancer based on protein- protein interaction network: Computational and in vitro analysis

**DOI:** 10.1016/j.bbrep.2026.102617

**Published:** 2026-05-21

**Authors:** Zakie Saadat, Seyed Mahdi Ahmadi, Mohammad Javad Bazyari, Seyed Hamid Aghaee-Bakhtiari

**Affiliations:** a Department of Medical Biotechnology and Nanotechnology, Faculty of Medicine, Mashhad University of Medical Sciences, Mashhad, Iran; b Bioinformatics Research Center, Basic Sciences Research Institute, Mashhad University of Medical Sciences, Mashhad, Iran

**Keywords:** Prostate cancer, Metastasis, System biology, Protein interaction network

## Abstract

Metastasis is a big challenge for prostate cancer patients that reduces their overall survival from 98.2% to 30%. Therefore, it is important to find the underlying mechanism driving metastasis and find a drug that can prevent cell invasion, migration, and metastasis. Here, we analyzed publicly available expression data of primary and metastasized tissue of Prostate cancer extracted from the GEO database. The obtained Differentially Expressed Genes (DEGs) were used to construct the Protein-Protein Interaction (PPI) and functional networks. These DEGs were enriched in pathways such as blood vessel development, and mesenchyme migration which are involved in the process of Prostate cancer metastasis. The hub genes in the constructed PPI network were subjected to the DrugBank query to find candidate drugs with potential inhibitory effects on metastasis. We found that Artesunate can target three hub genes: CSRP1, FLNA, and TPM1. Finally, we validate this finding by in vitro assessments on the LNCaP cell line. We observed that 2.5 mg/ml Artesunate in the scratch assay reduced the scratch closure rate by about 50% compared to the control in 48 h. These findings suggest that Artesunate may be a promising therapeutic agent for inhibiting prostate cancer metastasis.

## Highlights

• Analyzed expression data from prostate cancer tissues.
• Constructed PPI and functional networks using DEGs.
• Identified pathways involved in prostate cancer metastasis.
• Selected Artesunate targeting hub genes CSRP1, FLNA, TPM1.
• Artesunate (2.5 mg/ml) reduced scratch closure by ∼50% in 48h.

## 1 Introduction

Prostate cancer is the second most common cancer after lung cancer in men and the fifth deadliest cancer in the world.with nearly 1.5 million new cases and 400,000 deaths per year. The overall 5-year survival of prostate cancer patients is 98.2%, and when it becomes a metastasis, the survival rate decreases to 30%, although it is still a little high, but the main cause of death of these patients can be considered metastasis [1]. Screening and predicting methods such as PSA serum levels and the Gleason score are still remain imprecise and imperfect methods [2]. Unfortunately, some patients exhibit rapid resistance to standard androgen deprivation therapy (ADT) combined with/without microtubule-targeted taxane chemotherapy and leading to death [3]. Generally, α-receptor blockers and 5α-reductase inhibitors are prescribed for the treatment of prostate cancer [4].

Given the high costs and lengthy timelines associated with drug discovery, alongside the extensive availability of existing drugs and their known mechanisms of action [5], there is a significant opportunity to repurpose these available medications for new therapeutic uses [6]. This approach necessitates the ability to predict the potential effects of these drugs on the target disease, which can be achieved through a thorough understanding of the underlying biological mechanisms of the disease in question [7]. One of the approaches that helps to understand the disease mechanism in a general framework is systems biology [8]. System biology, relying on a holistic view and modeling, tries to understand the why of biological phenomena. One of the modeling methods in biological system is network modeling such as protein interaction network. Considering the high-yield technologies such as microarray and RNA sequencing and gathering expression data for different diseases, the system biology method is considered an efficient method to understand the process of metastasis in Prostate cancer. The aim of this study is to find the available drug to prevent the process of metastasis in Prostate cancer by using the expression data of primary Prostate cancer tissues and metastasized tissues and by network modeling method in system biology [9].

The development of high-throughput methods (such as large-scale microarrays and whole-genome sequencing) and bioinformatics methods has provided a wealth of information to guide drug screening. The discovery of hub genes and subsequent screening for candidate biomarkers or therapeutic targets has been applied in various cancer [10].

In 2019, Kim IW et al. published the article “Screening of drug repositioning candidates for castration resistant prostate cancer” and predicted the following drugs: sorafenib, olaparib, elesclomol, tanespimycin, ponatinib. The genes related to this disease such as MYL9, E2F2, APOE, ZFP36 were predicted to be reversed by these drugs, and in addition, it was predicted that lenalidomide in combination with pazopanib had the strongest effect on mCRPC [11].

In 2018, Turanli B et al. published the review study titled “Drug repositioning for effective prostate cancer treatment. Frontiers in physiology “. 25 drug candidates were introduced, 15 of which have undergone clinical trials, of which to date only zoledronic acid has been clinically approved for treatment as a non-cancer drug. In addition, the anticancer activities of the drugs mentioned in this study cover diverse and well-known mechanisms in this cancer, such as inhibition of mTOR and VEGFR2 signaling, inhibition of PI3K/Akt signaling, selective inhibition of COX and COX-2, inhibition of NF-kB pathway, inhibition of Wnt/b-Catenin, inhibition of DNMT1 and GSK-3b. In addition, it was shown that combination treatments with these drugs increase their effectiveness and reduce side effects [12].

Therefore, in the present study, expression data were searched in the GEO database and then analyzed, in the GEO2R environment, the differentially expressed genes were used to construct the protein-protein interaction network. String database and CluePedia tool were used for this purpose. Also, ClueGO tool was used to construct a functional network to investigate the pathways and functions in which the obtained genes are involved. Next, to find the key genes of the metastasis process, the key proteins of the network were found by different centrality algorithms. Then the key genes were searched in the DrugBank database and the appropriate drug was selected. Then, in the in vitro phase and on the LNcap cell line, the non-cytotoxic concentration of the desired drug was found with the MTT assay test. Then, the scratch assay was used to investigate the effect of the obtained drug on the mobility of Prostate cancer cells.

## 2 Materials and methods

### 2.1 Computational analysis

#### 2.1.1 Data source

Prostate cancer datasets and associated series were retrieved from the Gene Expression Omnibus (GEO) database (URL: https://www.ncbi.nlm.nih.gov/geo). A systematic analysis was performed utilizing the data aggregated from GEO and the NCBI Gene database. To maximize sample size and capture clinically relevant heterogeneity in prostate cancer, we used the publicly available GSE6919 dataset, which includes samples profiled on multiple microarray platforms (GPL8300, GPL92, GPL93).

Specifically, the GSE6919 gene expression profiles comprises a total of 271 Prostate Cancer (PCa) samples and 233 normal prostate tissue samples, integrating data from subsets GSE6604, GSE6605, GSE6606, and GSE6609 across three microarray platforms: GPL92, GPL93, and GPL8300.Cumulatively, it includes 504 samples consisting of 196 primary prostate tumor tissues and 75 metastasis tissues from 107 patients. More detail about the samples is presented in Table 1 [13].

**Table 1:** GSE6919 sample scope.

	GPL92	GPL93	GPL8300
Number of samples	167	164	171
Primary tumor tissues	66	65	65
Metastatic tissues	25	25	25

#### 2.1.2 Data processing and differential expression analysis

To identify genes important for prostate cancer metastasis, differential expression analysis (DEA) was performed using non-metastatic primary prostate tumor samples as the control group for comparison against metastatic tissues. For this purpose, after force normalization, Differential gene expression analysis for each platform was performed using the GEO2R online tool.

• Specify a GEO Series accession and a Platform
• Click 'Define groups' and enter names for the groups of Samples plan to compare, e.g., test and control
• Assign Samples to each group. Highlight Sample rows then click the group name to assign those Samples to the group. Use the Sample metadata (title, source and characteristics) columns to help determine which Samples belong to which group.
• Click 'Analyze' to perform the calculation with default settings.

Genes with adjusted p-value <0.05 and absolute logarithmic fold-change (logFC) > 1 were flagged and considered as metastasis-related DEGs.

The results integrated after separate analyses of each platform because of batch effect we could not merge the data before analyzing it one by one. After analysis, we merged the data to reduce the rate of false positives.

#### 2.1.3 Construction and analysis of protein-protein interaction network

The Cytoscape plugin, CluePedia was used to create a protein-protein interaction network. To improve network connectivity, additional high-confidence interactors were added (STRING score >0.8). Due to some genes being removed during tissue-specific gene removal, a maximum of 2 neighbors for each node were allowed to be included in the network. ClueGO the twin plugin of CluePedia, was then used to identify the functional roles and signaling pathways of the network nodes. The Biological Processes Gene Ontology (BP-GO) and Kyoto Encyclopedia of Genes and Genome (KEGG) databases were utilized as resources with p-value threshold less than 0.05 was chosen, and the minimum number of genes required for enrichment was set at three. Finally, ClueGO was used to construct and visualize the functional network within the Cytoscape environment.

#### 2.1.4 Hub genes identification and drug query

As the main goal of the current study, we were interested in finding key player genes in the Prostate cancer metastasis process and candidate drugs to cope with this phenomenon. In this regard, the top 15% genes according to three network centrality measurements, namely Degree, Betweenness, and Closeness were chosen as initial candidate hub genes. Furthermore, as the second criterion, we only included genes with average |logFC| > 1 for drug query. Drugbank database was utilized to find approved drug for the final candidate targets.

• For hub proteins exhibiting up-regulation (logFC>1) only, inhibitory drugs targeting these proteins were selected.
• for hub proteins exhibiting down-regulation (logFC<−1), only activating (agonistic) drugs were prioritized.

### 2.2 Experimental preparation

#### 2.2.1 Cell culture

The LNCaP cell line is an epithelial cell line derived from a human prostate carcinoma was acquired from the Pasteur Institute of Iran (Tehran, Iran). Cells were routinely maintained in Dulbecco's Modified Eagle's Medium (DMEM) (Gibco, Thermo Fisher Scientific, USA), supplemented with 10% (v/v) heat-inactivated fetal bovine serum (FBS; Gibco) and 1% (v/v) penicillin-streptomycin (100 U/mL penicillin and 100 μg/mL streptomycin). Cultures were kept in a humidified incubator at 37 °C with 5% CO_2_. Cells were passaged at 80-90% confluence using a 0.25% trypsin-EDTA solution (Sigma-Aldrich, USA).

#### 2.2.2 Cell viability assessment

The cytotoxicity of Artenimol (Zazzee.CO, USA) was evaluated using a 3-(4,5-dimethylthiazol-2-yl)-2,5-diphenyltetrazolium bromide (MTT) assay. Briefly, LNCaP cells were seeded into 96-well flat-bottom plates at a density of 7000 cells per well in 100 μL of complete growth medium and allowed to adhere overnight. Subsequently, the medium was replaced with fresh medium containing various concentrations of Artenimol (0.31, 0.62, 1.25, 2.5, 5.0, 10.0 and 20.0 μg/mL). four repetitions were considered for each concentration. After incubation for 24, 48, and 72 h, 10 μL of MTT reagent (5 mg/mL in phosphate-buffered saline (PBS)) was added to each well and the plates were incubated for an additional 4 h at 37 °C. The medium was then carefully aspirated, and 100 μL of dimethyl sulfoxide (DMSO) was added to each well to solubilize the formed formazan crystals. The plates were shaken for 10 min at room temperature. The absorbance of the solubilized formazan was measured at a wavelength of 570 nm, with a reference wavelength of 630 nm to subtract background noise, using a microplate reader. Cell viability was expressed as a percentage relative to the control group.

#### 2.2.3 Wound healing assay

The migratory capacity of LNCaP cells in response to Artenimol was assessed using a wound healing assay. Cells were seeded into 24-well plates at a density of 150,000 cells per well in complete growth medium (DMEM with 10% FBS) and incubated until they reached 80% confluence. A sterile p200 pipette tip was used to create a scratch wound in each well. The cellular debris was removed by washing twice with PBS. The cells were then incubated in low-serum medium (DMEM with 1% FBS) containing 2.5 μg/mL Artenimol or a control medium. Three repetitions were done for each of these groups. Images of the wound areas were captured immediately after scratching (0 h) and at 24 and 48 h post-scratch.

#### 2.2.4 Quantitative Real-Time PCR (RT-qPCR) for hub gene expression

##### 2.2.4.1 Cell line and treatment context

The expression levels of the identified hub genes were quantified in LNCaP (Lymph Node Carcinoma of the Prostate) cells following treatment with Artenimol. The LNCaP cell line, derived from a metastatic lesion of human prostatic adenocarcinoma, is characterized as being androgen-sensitive and possessing relatively low metastatic potential.

##### 2.2.4.2 RNA extraction and cDNA synthesis

Total RNA was extracted from treated and control cells using the TRIzol reagent, strictly following the manufacturer's protocol. Subsequently, complementary DNA (cDNA) was synthesized using a commercially available [Parstous Ultra cDNA Synthesis] kit.

##### 2.2.4.3 RT-qPCR protocol

Quantitative RT-PCR was performed utilizing the SYBR Green Master Mix on a Roche LightCycler 96-well system. The thermal cycling program was configured as follows: an initial denaturation step at 95∘C for 10 min, followed by 40 cycles consisting of 95∘C for 15 s (denaturation) and 60∘C for 1 min (annealing/extension). The specific primer sequences used for hub gene amplification are detailed in Table 2.

**Table 2:** The primers used in this study with their corresponding genes.

Gene	Forward	Reverse
TPM1	GCTCTCAGAAGGCAAATGTG	GACCTCTCCGCAAACTCAG
FLNA	TCAGGAGTCAGGGCTAAAGGTC	CGCACAGCATACTTATCTTGGTC
CSRP1	GGCGAGATTTACTGCAAAGGAT	GGCAGGGCTGACAGAGAAA

##### 2.2.4.4 Data analysis

Gene expression quantification was normalized against GAPDH, which served as the stable internal reference gene. Relative expression levels were calculated using the 2−ΔΔC method with primer efficiencies determined from standard curves and found to lie between 90% and 110%. A calibrator sample (e.g., untreated control) was used to compute fold changes relative to calibrator, and data are presented as relative expression (fold change) with statistical analyses described in Fig. 4D.

### 2.3 Statistical analysis

#### 2.3.1 Cell migration quantification

Quantification of cell migration from the captured images was performed by measuring the remaining scratch wound length using ImageJ/Fiji® software. then Statistical analysis using one-way ANOVA was performed to compare across treatment group with the untreated control group.

#### 2.3.2 Gene expression quantification

Relative gene expression data obtained from RT-qPCR analysis were processed using the REST (Relative Expression Software Tool) algorithm.

#### 2.3.3 Statistical significance

For experimental results, preliminary analysis involved checking for normal data distribution. After that, data were plotted as the mean ± standard error among independent replicates. Statistical analysis using one-way ANOVA was performed to compare across multiple treatment concentrations relative to the untreated control group. For all significant ANOVA results, Tukey's HSD post-hoc test was applied to adjust p-values for multiple comparisons. For all statistical tests, significance was indicated as *: p-value <0.05, **: p-value <0.01, ***: p-value <0.001.

## 3 Results

### 3.1 Differentially expressed genes in metastatic tissues

**Fig. 1:**
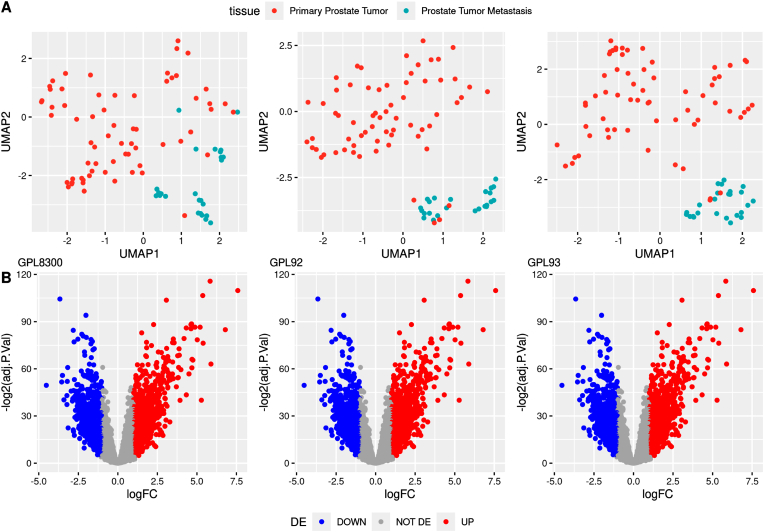
Umap plot and Volcano plot for GPL8300, GPL92 and GPL93. A: A UMAP plot was generated for each platform to visualize the distribution of the two study groups. Greater separation between the groups, accompanied by minimal overlap, indicates higher reliability and discriminatory power of the results. B presents a volcano plot constructed using the mean expression values of primary tumor and metastatic tissue across platforms, based on an adjusted *p*-value threshold of <0.05. In these plots, upregulated genes are represented in red, whereas downregulated genes are depicted in blue.

Of the 25,311 genes assessed in the GSE6919 dataset, which includes expression profiles from 196 primary prostate tumor tissues and 75 metastatic tissues, 24,934 were identified as tissue-specific and removed from downstream analysis. Differential expression was assessed between primary tumors and metastatic samples, to identify statistically significant genes, the following filtering criteria were applied separately on each platform, (adjusted P-value less than 0.05 (adj. P < 0.05), LogFC>+1 for Overexpressed Genes and LogFC<−1 for Under expressed Genes.) yielding three sets of differentially expressed genes (DEGs), with 827,77 and 181 genes identified in the GPL92, GPL93 and GPL8300 platforms, respectively. The lists of significant overexpressed and under expressed genes including 377 genes derived from the three initial output files were subsequently merged for downstream network and enrichment analyses. The specific results of the GEO2R analysis are provided below (Fig. 1). More information in the supplementary table.

### 3.2 Protein-protein interaction (PPI) and functional network

**Fig. 2:**
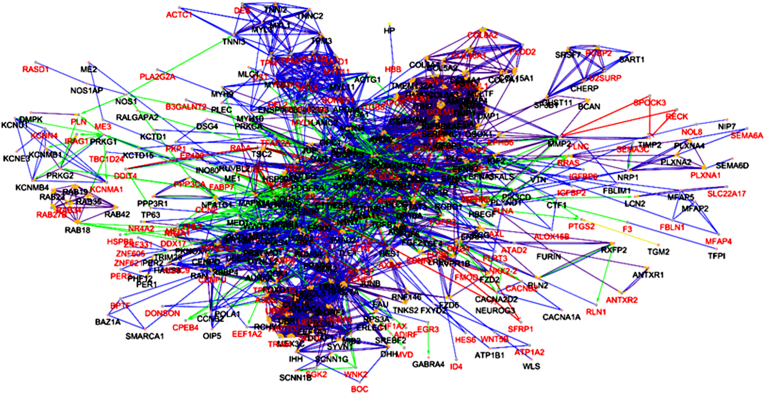
The protein–protein interaction (PPI) network was constructed using the CluePedia plugin. For PPI network construction, 377 differentially expressed genes were used to construct an initial network that was then expanded to achieve a biologically meaningful network. The final network included 466 nodes, of which 120 nodes were highly interconnected.

**Fig. 3:**
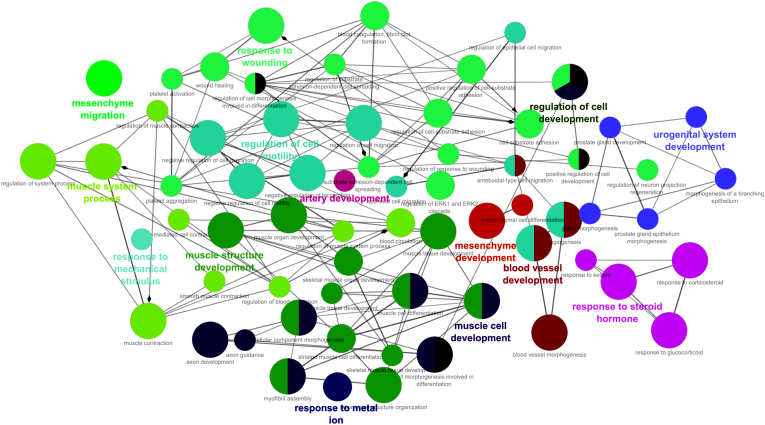
The functional network was constructed with The using the ClueGO plugin. pathway enrichment analysis revealed that the top enriched biological processes were mainly associated with cytoskeleton organization, cell adhesion, and migration-related signaling.

For PPI network construction, 377 differentially expressed genes were used to construct an initial network that was then expanded to achieve a biologically meaningful network. The final network included 466 nodes, of which 120 nodes were highly interconnected This connected network can be seen in Fig. 2. The pathway enrichment analysis revealed that the top enriched biological processes were mainly associated with cytoskeleton organization, cell adhesion, and migration-related signaling. These pathways are functionally linked to the identified hub genes TPM1, FLNA, and CSRP1, which play crucial roles in maintaining cell structure, motility, and metastatic potential. The integration of these findings suggests that dysregulation of these genes may contribute to the enhanced migratory behavior observed in metastatic prostate cancer cells. to cancer progression and invasion, functional enrichment assessment emphasizes the importance of these genes in the metastasis process. The complete functional network is depicted in Fig. 3.

**Fig. 4:**
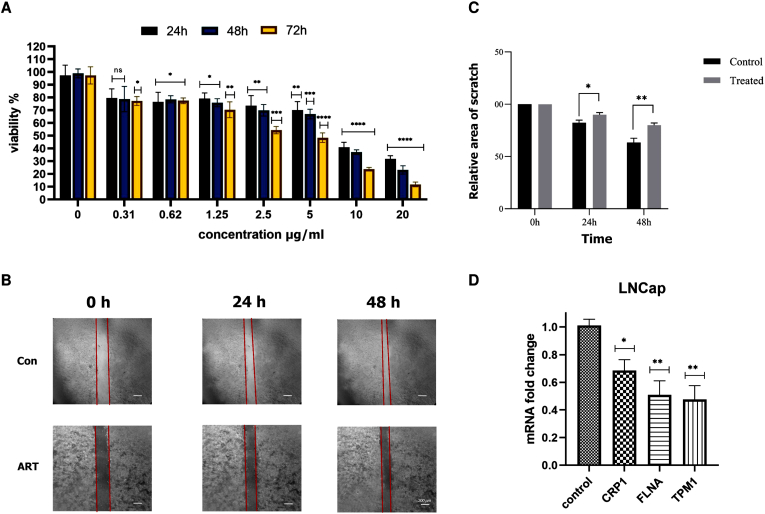
(A) Bar chart representing the results of the MTT cytotoxicity assay at 24, 48, and 72 h four repetitions were considered for each concentration. The graph illustrates the viability of LNCaP cells exposed to different concentrations of artesunate across the three time points. Statistical differences in cell survival between each treatment concentration and the untreated control group are indicated by ASTERISKS (For all statistical tests, significance was indicated as *: p-value <0.05, **: p-value <0.01, ***: p-value <0.001.). Error bars represent the standard deviation of replicate measurements within each group. from the concentration of 5 μg/ml, cell viability decreased significantly (pvalue = 0.0001). Also, the IC50 was determined at different times: IC50(24h) = 7.192 μg/ml, IC50(48h) = 4.757 μg/ml, IC50(72h) = 1.684 μg/ml (B) Representative images of the scratch assay at 0, 24, and 48 h, compared with the control group. three repetitions were done for each of these groups. (C) Quantitative analysis of the scratch assay performed using GraphPad Prism version 6, demonstrating that artesunate inhibited cell migration at 24 and 48 h. The results of one-way ANOVA analysis show the changes in wound closure rate at three times: 0, 24 and 48 h. These changes were compared with the control group using Prism GraphPad Prism v6 software, and a significant difference between wound closure in the treatment and control groups is observed, (D) Relative expression levels of TPM1, CSRP1, and FLNA genes in artesunate-treated cells the change is calculated compared with the control group by REST algorithm. These results show that the effect of Artesunate drug on LNCaP cells and their metastasis behavior is effective, and by reducing the expression of genes involved in metastasis, it can prevent cell invasion and tumor metastasis. Error bars represent the standard deviation of the mean (SD) across 40 biological replicates. The observed variability may reflect biological heterogeneity, RNA quality and RT-qPCR technical variability.

### 3.3 Hub genes and drug query

As a scale-free network with a top minor group of nodes with the highest topological importance, the constructed network was analyzed to find such hubs. Based on each centrality measurement, the top 15% nodes were selected as hub genes. These genes and their corresponding centrality measures can be found in Table 3. Cumulatively, 33 hub genes were found in the network as some of them were in the top 15% based on more than one centrality measurement. As it can be seen in Fig. 2, these 33 genes are highly interconnected and constitute a local dense community in the original network. Consequently, seven hub genes that had mean logFC larger than one were subjected to drug query in the DrugBank database. These hub genes, their corresponding logFC, and the result of this query have been provided in Table 4. Accordingly, we found Artenimol and Zinc salts.

**Table 3:** 33 hub genes were identified using combined centrality thresholds.

closeness hub	closeness centrality	betweenness hub	betweenness centrality	degree hub	degree centrality
S100A8	1.000000	GSTM2	0.166667	FGG	44
ME3	1.000000	GSTM4	0.166667	FN1	44
CSRP1	1.000000	KRT5	0.166667	UBE2C	37
GLUD2	1.000000	AR	0.031131	MYL9	36
AKR1C3	1.000000	FN1	0.022844	IGF1	34
MAOB	1.000000	TPM1	0.022270	TPM1	33
PDE5A	1.000000	FGG	0.021918	MYH11	32
ATP1A2	1.000000	APOE	0.017243	APOE	31
RAB27B	1.000000	HBB	0.016385	UBE2Z	30
AZGP1	1.000000	FLNA	0.015037	VCAN	28
ACP3	1.000000	FOS	0.01441	SPP1	27

**Table 4:** Hub genes and DrugBank database query result.

Drug	mean LFC	Gene
Artenimol	2.466474	TPM1
	2.678756	FLNA
	3.086534	CSRP1
Zinc Zinc chloride Zinc acetate Zinc sulfate	−2.033000	APOE
	−2.718200	FN1
	−2.132180	FGG
	3.703653	KRT5
	−3.501800	HBB
	−2.387650	S100A8

Artesunate was prioritized based on literature support for anti-metastatic activity. Strong evidence supports its anti-cancer effect, better drug-likeness and it can simultaneous targeting of multiple upregulated hub genes, Artesunate prevents cancer metastasis by reducing the expression of genes that play a key role in angiogenesis.

### 3.4 Effect of artesunate on LNCaPs cells’ migration

To find Artesunate non-cytotoxic concentrations, an MTT assay was carried out. As the bar chart in Fig. 4A shows, from the concentration of 5 μg/ml, cell viability decreased significantly (pvalue = 0.0001). Also, the IC50 was determined at different times: IC50(24h) = 7.192 μg/ml, IC50(48h) = 4.757 μg/ml, IC50(72h) = 1.684 μg/ml.

Next, scratch assay was performed at 2.5 μg/ml Artesunate. After 24 h, there was no significant reduction in cells’ mobility. But, after 48 h, the wound area of the control group closed more than Artesunate group. Images of scratched areas are illustrated in Fig. 4B. As indicated in Fig. 4C, two-way ANOVA analysis show the changes in wound closure rate at three times: 0, 24 and 48. These changes were compared with the control group and provide (p-value <0.05, for treated vs control at 24 h; p-value <0.01 at 48 h).

### 3.5 Artesunate reducing the expression of TPM1, CRPC1, and FLNA1 genes

The expression of the hubs obtained from the network analysis and confirmed in their impact studies were measured by Real-Time PCR in two treatment and control groups, increasing the expression of TPM1 is involved in cytoskeletal organization, cell proliferation, tissue invasion and metastasis. Increased expression of FLNA is associated with tissue invasion and suppression of apoptosis. Increased expression of CSRP1 is a key gene linked to resistance to treatment in metastatic prostate cancer. Fig. 4D shows the expression ratio between the control and treatment groups, mRNA fold change is calculated relative to control group. According to the statistical analysis, genes show different expression in the treatment group compared to the control group. These results show that the effect of Artesunate drug on LNCaPs cells and their metastasis behavior is effective, and by reducing the expression of genes involved in metastasis, it can prevent cell invasion and tumor metastasis.

## 4 Discussion

Prostate cancer, with nearly 1.5 million new cases and 400,000 deaths annually, is the second most common cancer in men and the fifth deadliest cancer worldwide. Several risk factors, including genetic mutations and changes in miRNA expression and androgen receptors, are associated with the onset and progression of prostate cancer. The accumulation of these mutations can lead to altered cell functions and ultimately malignant tumors. Uncontrolled growth is a key characteristic of malignant tumors, and in advanced stages, cancer cells can spread to secondary organs. Despite advancements in screening and treatment, metastasis significantly impacts overall patient survival. The discovery of new drugs is a time-consuming and costly process, but drug repositioning is recognized as an alternative approach that can reduce development costs and side effects, as the safety of repurposed drugs is already established in humans and clinical models [14].

The current therapeutic landscape for advanced prostate cancer relies on several key regimens. Androgen Deprivation Therapy (ADT) serves as the first-line treatment by reducing androgen levels to inhibit tumor growth, primarily targeting the androgen receptor (AR) signaling pathway crucial for prostate cancer cell proliferation. For later stages of metastatic prostate cancer (mPC), chemotherapy, such as Docetaxel, is often employed to disrupt microtubule function, leading to cell cycle arrest and apoptosis [15]. Targeted therapies, including Enzalutamide and Abiraterone, directly inhibit AR signaling or androgen production; Enzalutamide blocks AR translocation and DNA binding, while Abiraterone inhibits CYP17, thereby reducing testosterone synthesis. Additionally, Radium-223, a radiopharmaceutical, is utilized for bone metastases by delivering targeted radiation to induce DNA damage in cancer cells [16].

In contrast, Artesunate operates through distinct mechanisms. It generates Reactive Oxygen Species (ROS), inducing oxidative stress and cellular apoptosis, a different approach from conventional therapies focused on hormonal pathways or microtubule dynamics. Artesunate also activates intrinsic and extrinsic apoptotic pathways, potentially circumventing resistance mechanisms developed by prostate cancer cells against ADT and chemotherapy. Furthermore, its effects on cytoskeletal proteins may reduce cancer cell migration and invasion, addressing a critical challenge in metastatic prostate cancer (mPC) [17,18].

The potential advantages of Artesunate include overcoming resistance, as it offers a novel method to induce apoptosis via ROS, independent of androgen receptor signaling. Its potential for combination therapy is also significant; it could enhance the efficacy of existing treatments. For example, by increasing oxidative stress, it might sensitize cancer cells to chemotherapeutics like docetaxel, which may be less effective in resistant tumors. Artesunate may also exert differential effects on the tumor microenvironment, potentially influencing immune responses and tumor vasculature [18,19].

From a clinical perspective, exploring the efficacy of artesunate in combination therapies through clinical trials is crucial for understanding its potential in treating mPC. Developing biomarkers to predict response to artesunate could enable personalized treatment strategies. Further mechanistic studies are also warranted to elucidate the specific pathways influenced by artesunate in prostate cancer cells, thereby enhancing our comprehension of its role and effectiveness in combination therapies.

In summary, comparing artesunate with existing therapeutic regimens for metastatic prostate cancer reveals unique mechanistic pathways that may provide benefits in overcoming resistance and enhancing treatment efficacy. Future research should focus on elucidating these mechanisms and evaluating the therapeutic potential of artesunate in clinical settings, particularly as part of combination therapies.

In this study, our goal was to identify suitable drugs to prevent metastasis using computational analysis based on expression data and bioinformatics methods. We initially used the GEO database to locate relevant datasets, leading us to the GSE6919 microarray data. Next, we identified differentially expressed genes (DEGs) through differential expression analysis and used pathway enrichment analysis to uncover significant pathways associated with these genes. We then constructed a protein-protein interaction (PPI) network and utilized its analysis to identify key genes for drug targeting. A total of 33 hub genes were selected based on centrality criteria (closeness, betweenness, and degree). Hub genes with |logFC| > 1 were considered suitable drug candidates, and we searched for corresponding drugs in the DrugBank database. Activating drugs were identified for downregulated hub genes, while inhibitory drugs were identified for upregulated hub genes, with only FDA-approved drugs being considered.

Finally, we arrived at two drugs, Zinc and Artesunate, Artesunate was prioritized based on literature support for anti-metastatic activity. Strong evidence supports its anti-cancer effect, better drug-likeness and it can simultaneous targeting of multiple upregulated hub genes, Artesunate prevents cancer metastasis by reducing the expression of genes that play a key role in angiogenesis**.** Artesunate treatment significantly reduced the migration of LNCaP cells compared to the control group. Based on the integrated computational and experimental findings, we propose a potential mechanism of action for Aresunate in metastatic prostate cancer. Artesunate may exert its anti-metastatic effects by modulating cytoskeletal remodeling through TPM1, regulating cell adhesion and signaling via FLNA, and influencing smooth muscle differentiation and proliferation through CSRP1. These interactions could collectively suppress cell migration and invasion, providing a mechanistic explanation for the observed inhibitory effects in vitro.

TPM1 (Tropomyosin 1) is recognized for its role in muscle contraction regulation and cytoskeletal organization. Artesunate affect TPM1 expression, acting as a tumor suppressor. Increased expression of TPM1 plays a role in cell proliferation, tissue invasion, and metastasis [20].

FLNA1 (Filamin A) plays a crucial role in cellular structure and motility. Evidence suggest that artesunate could affect FLNA1 expression, possibly influencing metastasis and cell migration. Increased expression of FLNA is associated with tissue invasion and suppression of apoptosis [21].

CRPC1 (Castration-Resistant Prostate Cancer 1) is a key gene associated with resistance to treatment in metastatic prostate cancer. Studies suggest that Artesunate influences CRPC1 expression through signaling pathways or by modulating microRNAs. Increased expression of CSRP1 is associated with tissue invasion and tissue proliferation [22].

In 2011, FRANS HERWIG JANSEN et al. found that Artesunate decreased the expression of P53 and EGFR, which ultimately increased the survival of patients with advanced cervical cancer [23].

Li, Yue et al., in 2020, in a review study on the keyword Artemisinin and its derivatives reviewed the available articles up to May 2019 and reported: In preclinical studies, ARMs can progress by inducing cell cycle arrest and apoptosis. Prevent tumors and suppress metastases by inhibiting EMT and angiogenesis. Some clinical trials support Artemisinins as promising candidates for cancer treatment [24].

These findings highlight the potential of Artesunate as a repositioned drug for metastatic prostate cancer.

The observed downregulation of TPM1, FLNA, and CSRP1 by Artesunate could reflect regulation at multiple regulatory layers (a) Artesunate may modulate signaling pathways (e.g., NF-κB, PI3K/AKT/mTOR, and MAPKs). that govern transcription factors targeting TPM1, FLNA, and CSRP1; ROS-activated kinases such as p38/JNK can alter transcription factor activity and chromatin accessibility, contributing to transcriptional repression of cytoskeletal genes [[25], [26], [27]]. (b) Epigenetic alterations (DNA methylation and histone modifications) at promoter/enhancer regions could further suppress transcription by changing chromatin state and promoter activity [28]. MicroRNA–mediated regulation (e.g., putative miR-XX/miR-YY) could directly reduce transcript stability or translation of TPM1, FLNA, and CSRP1, consistent with established roles of miRNAs in post-transcriptional control of cytoskeletal genes [29,30]. (d) Post-transcriptional or post-translational regulation via proteostasis may also contribute, as cellular stress responses can rewire RNA-binding protein activity and protein turnover, shaping the net output of these transcripts and proteins [31]. To test these hypotheses, we recommend TF-binding analyses and promoter/chromatin studies (promoter-reporter assays; ChIP-qPCR for NF-κB, AP-1, p53, and relevant histone marks), genome-wide and targeted miRNA profiling with 3′UTR reporter validation, promoter-reporter assays, and pathway inhibition experiments (e.g., p38/JNK inhibitors, PI3K/AKT/mTOR modulators, MRTF-SRF and RhoA/ROCK axes) to assess rescue of TPM1/FLNA/CSRP1 expression; additional assays could include mRNA stability analyses and proteostasis-focused investigations. Together, these approaches will illuminate whether transcriptional, epigenetic, post-transcriptional, or proteostatic mechanisms predominate, or act in concert, in Artesunate-mediated downregulation of these cytoskeletal genes.

This study has several limitations. First, the bioinformatics analysis was based on publicly available datasets, which may introduce bias and require further validation. Second, the in vitro experiments were conducted using only one prostate cancer cell line LNCaP, which may limit the generalizability of the findings. Third, molecular docking or simulation studies were not performed to confirm the direct interaction between Artesunate and the predicted target proteins and Absence of dose-response migration data. Future studies should include additional cell lines (e.g., PC-3, DU145), in vivo models, protein-level validation (Western blot), Single cell line validation to strengthen the conclusions.

## 5 Conclusions

System biology as a new approach in biology can help to target studies. Systems biology enables rational drug repurposing by integrating high-throughput data with computational modeling. Biomedical sciences, which rely on biological findings and try to treat diseases and solve problems and problems caused by them, can potentially benefit from the system biology. In this study, an attempt was made to evaluate one of the clinical challenges of prostate cancer, i.e. metastasis, and find a suitable drug solution for it by relying on biological data instead of reviewing the literature alone and using a systematic approach. This was done with the purpose of drug reuse and recovery to overcome the difficulties and challenges of new drug design and reduce its time and cost. In this study, by using graph theory and protein network modeling and finding important and key genes that have an effect on the disease control process, Artesunate drug for prostate cancer was recovered and introduced. Then by operating the in vitro phase and performing confirmatory tests, this drug was evaluated. This research has two important results:

1. Artesunate drug has the ability to inhibit cell migration and its testing in the animal phase is justified.
2. In general, a systematic and logical approach to life, regardless of laboratory technical issues, is considered a suitable and even obligatory method in biomedical sciences.

Overall, our integrative computational and experimental approach highlights Artesunate as a promising repositioned drug candidate for metastatic prostate cancer, warranting further mechanistic and preclinical investigations.

## Funding

This work was supported by 10.13039/501100004748Mashhad University of Medical Sciences, (Grant number 992318).

## CRediT authorship contribution statement

**Zakie Saadat:** Conceptualization, Data curation, Formal analysis, Investigation, Writing – original draft, Writing – review & editing. **Seyed Mahdi Ahmadi:** Conceptualization, Data curation, Formal analysis, Writing – review & editing. **Mohammad Javad Bazyari:** Data curation, Investigation, Validation, Writing – review & editing. **Seyed Hamid Aghaee-Bakhtiari:** Funding acquisition, Project administration, Supervision, Writing – review & editing.

## Declaration of competing interest

None declared.

## Data Availability

The data used in this study are publicly available through the GEO database.
